# Nitric Oxide in Plant Functioning: Metabolism, Signaling, and Responses to Infestation with Ecdysozoa Parasites

**DOI:** 10.3390/biology12070927

**Published:** 2023-06-28

**Authors:** Jakub Graska, Justyna Fidler, Marta Gietler, Beata Prabucka, Małgorzata Nykiel, Mateusz Labudda

**Affiliations:** Department of Biochemistry and Microbiology, Institute of Biology, Warsaw University of Life Sciences-SGGW, Nowoursynowska 159, 02-776 Warsaw, Poland; justyna_fidler@sggw.edu.pl (J.F.); marta_gietler@sggw.edu.pl (M.G.); beata_prabucka@sggw.edu.pl (B.P.); malgorzata_nykiel@sggw.edu.pl (M.N.)

**Keywords:** abiotic stress, biotic stress, nitric oxide, peroxinitrite, pest, phytopathogen, plant-animal interaction, post-translational modification, reactive nitrogen species, reactive oxygen species

## Abstract

**Simple Summary:**

Nitric oxide (NO) is a key molecule that has an important role in the plant life cycle. It mediates a range of physiological processes and responses to stresses (e.g., drought, salinity, or parasite invasion). Despite many studies, knowledge about NO involvement in these processes is incomplete. This review describes the influence of NO on physiological and biochemical processes and gene expression. It thoroughly discusses the interaction network of NO and other molecules in plant cells. Moreover, it highlights mechanisms of NO-dependent defense response against infestation with Ecdysozoa species, like nematodes, insects, and arachnids.

**Abstract:**

Nitric oxide (NO) is an important signaling molecule that is involved in a wide range of physiological processes in plants, including responses to biotic and abiotic stresses. Changes in endogenous NO concentration lead to activation/deactivation of NO signaling and NO-related processes. This paper presents the current state of knowledge on NO biosynthesis and scavenging pathways in plant cells and highlights the role of NO in post-translational modifications of proteins (S-nitrosylation, nitration, and phosphorylation) in plants under optimal and stressful environmental conditions. Particular attention was paid to the interactions of NO with other signaling molecules: reactive oxygen species, abscisic acid, auxins (e.g., indole-3-acetic acid), salicylic acid, and jasmonic acid. In addition, potential common patterns of NO-dependent defense responses against attack and feeding by parasitic and molting Ecdysozoa species such as nematodes, insects, and arachnids were characterized. Our review definitely highlights the need for further research on the involvement of NO in interactions between host plants and Ecdysozoa parasites, especially arachnids.

## 1. Introduction

Nitric oxide (NO) is a lipophilic molecule involved in signal transduction in cells, and its biosynthesis occurs endogenously in plants. NO belongs to the group of reactive nitrogen species (RNS), which are known to play many roles in the regulation of plant physiological processes [1,2,3,4]. NO is involved in seed germination, root development, flowering, fruit ripening, senescence, stomatal movement, and photosynthesis [4,5,6,7]. It is also involved in plant responses to abiotic and biotic stresses [2,8,9,10]. Nevertheless, among the available data from previous years, the most analyzed topic was the role of NO in the regulation of plant growth and development. NO, like other RNS, has a stimulating effect in low concentrations, while in higher amounts, it inhibits metabolic processes [4]. The multifunctional role of NO is related to its half-life time. At a low concentration, NO has a longer half-life time compared to a higher concentration. In the first case, NO can diffuse in tissues for minutes (even hours) over long distances, which enables regulation of physiological processes. On the other hand, at locally higher concentrations (short half-life time), metabolic processes are inhibited [11,12].

Participation of NO in physiological process is merely a part of precise combination of external and internal signals. Seed germination is strictly regulated by a number of factors, including NO [13]. Experimental data from recent years show that NO certainly stimulates this process. For a long time, seed companies used nitrates and nitrites to promote the germination of dormant seeds, due to induction of NO synthesis by the availability of these compounds in tissues [13,14]. Subsequent analysis showed involvement of several nitrogen-containing compounds in breaking seed dormancy (nitrate, nitrite, hydroxylamine, azide, sodium nitroprusside (SNP)) [4,15]. Application of NO in various concentrations is able to break seed dormancy depending on plant species. NO can also induce root development and formation: primary root, lateral, and adventitious [13]. Moreover, NO also plays a role in senescence. It was found that changes in local concentration of NO could delay or alleviate senescence [12,16,17]. The main linking of NO and other factors involved in pro-senescence activity is H_2_O_2_. Increasing accumulation of both of these components leads to protein and lipids oxidation. This event promotes programmed cell death (PCD) and activation of expression of PCD-related genes [18,19]. On the other hand, the impact of ROS might be lowered through NO participation in the increased activity of antioxidant enzymes [20].

The basic factors related to the perception of NO by plant cells are its synthesis and catabolism. To date, several NO production and capture reactions have been described in enzymatic and non-enzymatic pathways [21]. Moreover, it should be mentioned that NO is involved in protein post-translational modifications (PTMs); therefore, it contributes to plant responses to internal and external stimuli [22,23,24]. NO-mediated PTMs and metabolic pathways are regulated by NO crosstalk with other signaling molecules such as reactive oxygen species (ROS) and phytohormones. Changes in the amounts of those signaling molecules contribute to the regulation of plant growth and development and/or stress responses [25]. In this review, we present the current state of knowledge on NO metabolism and its relationship with ROS, RNS, and phytohormones. In addition, we attempted to summarize findings regarding the poorly understood role of NO in plant responses to attack and feeding by various herbivorous Ecdysozoa parasites.

## 2. Biosynthesis of NO in Plants

**Figure 1:**
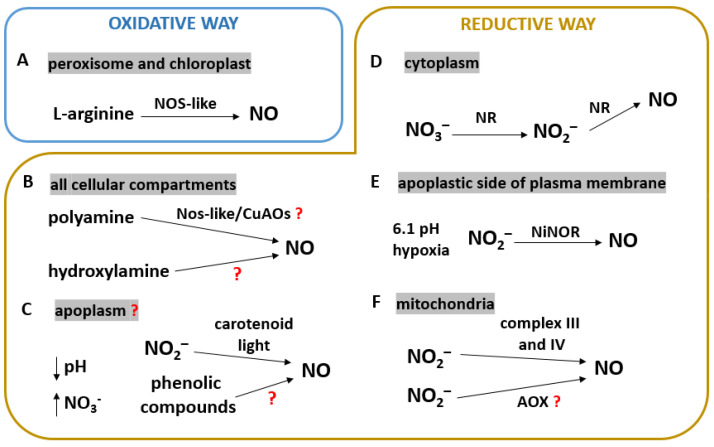
Nitric oxide (NO) synthesis pathways in plants. (**A**) L-arginine-dependent synthesis via nitric oxide synthase (NOS)-like activity. (**B**) NO production via polyamine transformation (NOS-like activity/copper amine oxidases (CuAO) are likely involved). (**C**) Non-enzymatic NO production: reduction of nitrites by carotenoids in the presence of light; putative reduction of phenolic compounds to NO. (**D**) NO production via double reduction of nitrogen species (in the order of nitrate (NO_3_^−^) and nitrite (NO_2_^−^)) by nitrate reductase (NR). (**E**) Reduction of NO_2_^−^ to NO by NiNOR (membrane-bound nitrite reductase) under hypoxic conditions and slightly acidic pH. (**F**) Electron transport chain-dependent NO production by complexes III and IV and putative NO generation by mitochondrial alternative oxidase (AOX). See text for details.

The presence of NO in higher plants has been well-documented in the literature over the last 40 years of research. However, the most unknown issues are NO production and signaling. Several biosynthetic pathways in plants have been described so far. The most speculated pathways of NO biosynthesis in plants are the reduction and oxidative pathways [26] (Figure 1).

### 2.1. NOS-like Activity

The oxidative pathway of NO synthesis that occurs under normal conditions is still not fully understood. It presupposes the existence of several homodimers of an enzyme called nitric oxide synthase (NOS) [27,28]. Works by Bredt and Snyder [29] and Stuehr et al. [30] reported that NOS activity in mammals was accompanied by double monooxygenation of L-arginine to NO and citrulline. More specifically, NOS uses two co-substrates, molecular oxygen and nicotinamide adenine dinucleotide phosphate (NADPH), to catalyze the oxidation of the guanidine nitrogen of L-arginine. The intermediate product of five-electron oxidation is N-hydroxy-L-arginine [31,32]. The active human NOS isoforms are neuronal NOS (nNOS), endothelial NOS (eNOS), and inducible NOS (iNOS). Each of them has distinct characteristics [33]. In addition, nNOS and eNOS are known to be associated with NO signal-dependent cellular processes. In turn, the role of iNOS differs from other NOS. iNOS produces NO as a cytotoxic agent in immune response/pathological conditions. Nevertheless, these three enzymes were not fully identified in plants.

Potential NOS activity in plants was reported by two research groups in 1998. Durner et al. [34] showed increased NOS-like activity in *Nicotiana tabacum* infected with tobacco mosaic virus. In turn, the work of Delledonne et al. [35] attempted to determine the presence of NOS in *Glycine max* infected with *Pseudomonas syringae*. The results showed calcium-dependent NOS-like activity in the *G*. *max* cytosolic fraction. However, the researchers did not report plant NOS homologues in *G*. *max*. Only a protein NOS inhibitor has been identified. In the following years, many researchers tried to prove the activity of NOS in plants. It has been suggested that expression of the *A*. *thaliana NOS1* gene is essential for L-arginine-dependent NO biosynthesis [36]. Since then, several possible NOS-like enzymes have been proposed (Figure 1A); however, no conjectures have ever been confirmed. Previously described Arabidopsis NOS-like enzymes were eventually classified as GTPases and renamed Nitric Oxide-Associated 1 (AtNOA1). In addition, AtNOA1 has been recognized as a family of GTPases with a circular permutation (cGTPase) [37,38,39].

Data obtained by Jeandroz et al. [40] within the international multidisciplinary consortium 1000 Plants (1KP) brought a new perspective on the discussed issues. In this study, gene sequencing was performed to obtain phylodiversity data from over a thousand land plants and algae. Bioinformatics analysis did not confirm the presence of animal NOS homologues in plants. It has been suggested that the absence of NOS in higher plants is evidence of the loss of this enzyme during evolution.

Although the exact mechanism of the enzymatic production of NO from arginine remains elusive, studies provide new evidence of the involvement of NOS-like activity in regulating plant functions such as development [25,35,41,42,43], cadmium stress responses [44,45], responses to pathogens [6,46,47], and protection against UV-B radiation [48].

### 2.2. NO Synthesis via Polyamine and Hydroxylamine Pathways

Other proposed ways of producing NO under oxidative conditions are the polyamine- and hydroxylamine-mediated pathways [32]. Researchers pointed to a possible link between NO and polyamines (PAs) in plant responses to abiotic and biotic stresses [41,42,44,49]. The production of NO through the oxidation of PAs is related to the conversion of putrescine, spermine, or spermidine. These experimental data were obtained with *A*. *thaliana* plants [45,46,47]. However, there is no evidence of an enzyme that converts PAs into NO. Comparison of plant and animal PA oxidases shows different mechanisms of enzymatic catalysis. As a consequence, animal PA oxidases are not involved in NO production [41]. However, in plants, the interaction of NO and PAs in response to stress can cause the accumulation of osmoprotectants, e.g., proline and gamma-aminobutyric acid, during drought [50,51].

Researchers also speculated that in plants, copper amine oxidases (CuAO) might affect polyamine-mediated NO synthesis (Figure 1B). The work of Recalde et al. [44] systematized knowledge about the putative role of CuAOs in NO production. Data presented by Wimalasekera et al. [52] showed that the Arabidopsis *CuAO1* mutant (with the loss of the gene encoding this enzyme) accumulated less NO than wild-type (WT) plants under salinity. Researchers hypothesized that during PA-dependent NO production, NO regulated NOS-like activity and/or nitrate reductase (NR) activity. What is more, NR is also involved in the reductive pathway of NO synthesis. The following experiment was performed by Groß et al. [53] using Arabidopsis *CuAO8* knockout mutant. The mutants contained a lower concentration of NO than WT under salt stress and elicitor (INA—salcic acid analog) treatment. In this case, the lower NO content was not due to the inhibition of NR, because its activity was not changed. At the same time, *CuAO8* mutants showed high arginase activity. In addition, supplementation with arginine or an arginase inhibitor led to an increase in NO production. These data showed the role of CuAO8 in the production of NO via an arginase-dependent pathway.

Another putative pathway of NO biosynthesis is the oxidation of hydroxylamine to NO. Report by Rümer et al. [54] demonstrated the ability of tobacco cell suspension cultures to produce NO via hydroxylamine oxidation (under oxidative conditions) (Figure 1B). However, such hydroxylamine activity was not observed in intact plants.

### 2.3. Non-Enzymatic Production of NO

Knowledge about non-enzymatic NO synthesis is significantly limited. Reduction of nitrates to NO without enzymes is possible in specific conditions, such as a low pH and the presence of nitrates in the apoplasts [6,55,56]. NO synthesis may be induced by phenolic compounds (Figure 1C) as it was shown in aleurone layer of *Hordeum vulgare.* In addition, it has been proposed that altered content of abscisic acid (ABA) and gibberellin may affect NO production in germinating *H*. *vulgare* seeds. Another putative way of non-enzymatic NO production is the carotenoid-mediated reduction of nitrites to NO in the presence of light [56,57]. In the dark, carotenoids and nitrogen dioxide are converted into nitrosating agents, which can lead to nitrosative cell damage (Figure 1C). Nevertheless, this method of NO production is still controversial.

### 2.4. Role of NR in NO Synthesis

Nitrate reductase is a key enzyme involved in the assimilation and metabolism of nitrogen in plants. It is also the best-characterized enzyme involved in NO synthesis. As shown in Figure 1D, NR is a cytoplasmic enzyme that catalyzes the reduction of nitrate to NO. However, it is well known that NR primarily catalyzes the reduction of nitrate to nitrite. This reaction requires the availability of an electron donor—NADP—and the presence of coenzymes in the form of molybdopterin, hem, and flavin adenine dinucleotide (FAD). The next nitrogen fixation reaction is the reduction of nitrite to ammonia in the plastids by nitrite reductase (NiR). The active NR is present in a homodimeric complex [1,58,59]. Studies have shown that NR has an additional function of nitrite: NO reductase (Ni-NR activity) (NAD(P)H + 3 H_3_O^+^ + 2 NO_2_^−^ → NAD^+^ + 2 NO + 5 H_2_O) [27]. It can occur under certain conditions such as hypoxia, a highly acidic environment, and high nitrite concentration [58,60,61]. The *A*. *thaliana* genome contains two NR-coding genes: *NIA1* and *NIA2* [62]. In Arabidopsis, the expression of both is required for the formation of the active NR enzyme. Experiments with *A*. *thaliana* mutants of these genes revealed the essential role of NR in NO synthesis in various plant physiological processes such as stomatal closure [63,64], flowering [65], root development [66,67], and responses to biotic and abiotic stress factors [68,69,70].

The work of Tejada-Jimenez et al. [71] systematized the knowledge on the reduction of nitrate to NO in photosynthetic eukaryotes. It has been suggested that NR may catalyze another direct NO production reaction in the algae *Chlamydomonas reinhardtii* [72] through the interaction of NR with NO-forming NiR (NOFNiR). NOFNiR belongs to the Amidoxime Reducing Component (ARC). It cooperates with NR in the production of NO through nitrite reduction-dependent electron transfer from NAD(P)H to NO. NOFNiR activity is specific to normoxia. NR and NOFNiR are regulated by the altered gene expression of transcription factors and at the protein level [26,72]. However, to date there is no evidence of NOFNiR NO production in higher plants.

However, regulation of NO synthesis by NR is also possible through the pathway associated with truncated hemoglobin 1 (THB1), which was observed in *C. reinhardtii* [73]. The pathway is based on the reduction of THB1 by NR. It leads to the initiation of THB1 diaphorase activity in the presence of oxygen and the conversion of NO to nitrate. These data were confirmed using a *THB1* knockout mutant of *C*. *reinhardtii* under sulfur deprivation conditions. It was observed that NO synthesis was higher in the *THB1* mutant under sulfur deprivation than in the WT [74].

### 2.5. A Plasma Membrane-Bound Nitrite Reductase

The ground-breaking works [75,76] demonstrated alternative NO production in *N*. *tabacum* root extracts. A membrane-bound nitrite reductase (NiNOR) was identified, and its activity was associated with cytoplasmic NR. NiNOR is bound to the plasma membrane. The conversion of nitrite to NO required NAD(P)H as an electron donor. In vitro, methyl viologen and cytochrome electron sources have been shown to stimulate NO generation. Moreover, NO biosynthesis occurred on the apoplast side of the membrane (Figure 1E). The highest NiNOR activity was observed under hypoxic conditions. Moreover, the availability of nitrates and succinates influenced the reduction of nitrates to nitrites by apoplastic NR. Consequently, this was a limiting factor for NiNOR activity. In addition, the research showed that NiNOR was most active in an acidic environment (pH 6.1), which is similar to the apoplastic environment. It was suggested that NiNOR may be involved in the detection of nitrogen availability in the soil. In addition, the study of tobacco mycorrhiza showed the participation of NiNOR in the synthesis of NO [77].

### 2.6. NO Synthesis in Mitochondria

The reduction of nitrite to NO in the mitochondria can be carried out by the electron transport chain (ETC). ETC-dependent NO production was observed in the roots of *H*. *vulgare* [78]. Complexes III and IV were noted to be the main ETC sites involved in NO synthesis (Figure 1F).

In addition, there is evidence that mitochondrial alternative oxidase (AOX) is involved in NO production. It was assumed that increased AOX activity was accompanied by limited NO production in *N*. *tabacum* leaves under hypoxic conditions [79,80]. Mutant tobacco plants lacking the genes encoding AOX had lost the alternative route of the respiratory chain, and they were characterized by higher levels of mitochondrial O_2_^−^ and apoplastic NO compared to WT. Alternative oxidase is involved in the modulation of ROS and RNS concentrations in plant’s mitochondria. It has been suggested that the mitochondrial activity of AOX may reduce concentrations of ROS and RNS through limiting the leakage of electrons from the ETC. Thus, the production of ROS and RNS is inhibited by AOX [79].

Recent data point to two new roles for AOX in the NO metabolism depending on oxygen levels. It has been observed that in normoxia, AOX can capture NO, which may reduce the formation of peroxinitrite (ONOO^–^) and tyrosine nitration [81]. In addition, AOX activity under hypoxic conditions led to the production of NO through supporting phytoglobins (PGBs)—NO cycle (PGBs-NO). This has been observed in transgenic tobacco with *AOX* overexpression [82,83]. Alternative oxidase reduced tyrosine nitration through regulating PGB expression and NO production. Furthermore, under these conditions, the AOX-PGBs-NO triad interaction could support ETC (especially complex I) through promoting proton translocation. This AOX-mediated NO synthesis leads to the sustained production of ATP in the mitochondria [82,84,85].

## 3. NO Scavenging

**Figure 2:**
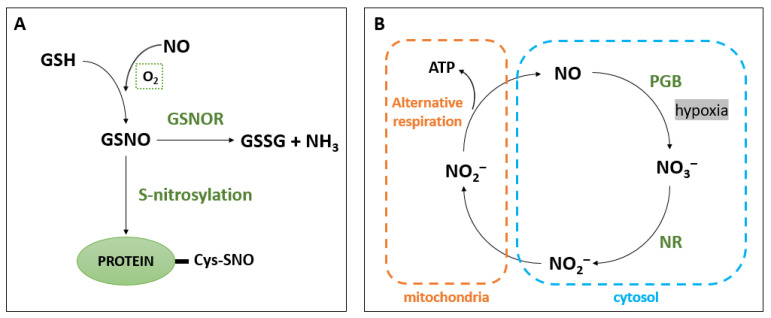
NO scavenging pathways in plant cells. (**A**) The interaction of reduced glutathione (GSH) and nitric oxide (NO) in the presence of light leads to the production of *S*-nitrosoglutathione (GSNO). In the next steps, GSNO can be converted to glutathione disulfide (GSSG) and ammonia (NH_3_). GSNO is involved in the *S*-nitrosylation of cysteine residues (Cys-SNO). (**B**) Phytoglobin (PGB)–NO cycle. Under hypoxic conditions, nitrate (NO_3_^−^) is reduced to nitrite (NO_2_^−^) in cytosol by nitrate reductase (NR). Then, NO_2_^−^ is transported to the mitochondrion, where it is converted to NO via an alternative respiration pathway, resulting in the production of a negligible amount of adenosine triphosphate (ATP).

To maintain adequate NO content, there must be a balance between NO biosynthesis and scavenging pathways. The reduction of NO content is related to enzymatic scavenging. One of the scavenging enzymes is S-nitrosoglutathione reductase (GSNOR), which belongs to the alcohol dehydrogenase (ADH) family [86,87,88]. GSNOR participates in the conversion of *S*-nitrosoglutathione (GSNO) to glutathione disulfide (GSSG) and ammonia (NH_3_) as well as protein S-nitrosylation (R-SNO) (Figure 2A). Moreover, the availability of GSNO depends on the interaction between NO and reduced glutathione (GSH) in the presence of oxygen. Consequently, low GSH content leads to the production of a stable form of NO—GSNO (endogenous low-weight *S*-nitrosothiol (SNO)). Accordingly, GSNO is recognized as a cellular source of NO and its transporting form [86,88,89]. It is assumed that a lack of GSNOR activity promotes the regulation of NO signaling through R-SNO of specific cysteine residues in proteins [90].

In addition, studies on Arabidposis demonstrated a possible mechanism of AtGSNOR1 autophagy with R-SNO under hypoxic conditions. Enzyme autophagy was enabled by S-nitrosylation of the Cys-10 residue in AtGSNOR1. In consequence, there were changes in GSNOR1 conformation, which led to the possible interaction of the autophagy machinery with the AUTOPHAGY-RELATED8 (ATG8) interacting motif [90]. These results suggest that NO-dependent PTMs were induced in the Arabidopsis mutant lacking R-SNO in GSNOR. Therefore, it is an example of GSNOR autoregulation at different oxygen contents. This mechanism can occur, for example, during transient hypoxia, such as in the initial phase of seed germination [91]. In the case of seed germination of *A*. *thaliana*, an increased concentration of GSNO was observed, accompanied by an increased expression of the *GSNOR1* gene. This was possible due to the simultaneous regulation of GSNOR and NO expression under hypoxia [90].

Furthermore, NO capture is regulated by PGBs (Figure 2B), previously known in plants as non-symbiotic hemoglobins [92,93]. Phytoglobins are hem-containing proteins. Their participation in NO homeostasis is related to the ability to bind NO and other diatomic molecules (e.g., oxygen). PGBs can be divided into four subfamilies, the symbiotic class and the other three non-symbiotic classes [27,94,95]. The first class, known as phytoglobins1 (Phytogbs1), has a high affinity for oxygen and the ability to capture NO molecules under hypoxic conditions [93,96]. Meanwhile, it has been observed that plants under anaerobic conditions used nitrite as an alternative electron acceptor for alternative respiration [97,98]. In this case, the phytoglobin (PGB)-NO cycle plays a significant role in the production of ATP and the reduction of NO content. First, hypoxia-induced PGB converts NO to nitrate, and in turn, nitrate is reduced to nitrite by cytosolic NR. Then, nitrite is transported to the mitochondria, where it acts as terminal electron acceptor. This reaction leads to the production of NO, which can diffuse back into the cytosol. Consequently, the available NO would be oxidized to nitrate, which can be a substrate for NR [96,99,100]. The PGB-NO cycle does not generate a large amount of energy stored in ATP [28,101,102]. Moreover, this cycle could participate in hypoxia in the production of NAD^+^ through fermentation [99]. It is also believed that the activity of this cycle requires the presence of cofactors for the conversion of nitrogen compounds. Thus, the PGB-NO cycle is involved in maintaining the balance of the amount of NADH/NAD^+^ and NADPH/NADP^+^ and the ratio of ATP/ADP [103,104,105].

The work of Armstrong et al. [106] presented a complex model of the response of root tissues to anoxia (in watery soil). The researchers hypothesized that PGB was involved in modulating NO and O_2_ levels in the roots surrounded by “an anaerobic core”. Thus, the PGB-NO cycle could scavenge excess reactive species and alleviate energy crises. Similar results were obtained in *H*. *vulgare* plants under water stress [107]. Researchers linked PGB’s regulation of NO to plant responses to stresses; for example, the activity of the PGB-NO cycle was also observed in legume nodules under drought and it participated in energy regeneration [108]. Nitrogenase, an enzyme involved in symbiotic nitrogen fixation (SNF), is sensitive to the presence of O_2_. Therefore, legume nodules are kept in low-oxygen conditions by leghemoglobin [108,109]. An increasing amount of NO has also been observed under hypoxic conditions, and it triggered PGB-NO respiration. This interaction helps to maintain the energy status in the nodules during drought.

On the other hand, the work of Kumari et al. [110] proposed a probable role of the PGB-NO cycle in the mycorrhizal symbiosis of plants. *Phytogbs1* was hypothesized to play a role as a regulator of NO homeostasis, which enabled the symbiotic colonization of roots and the recognition of potential pathogenic microorganisms. The PGB-NO cycle regulates the concentration of NO in plant tissues not only under stress conditions, but also in physiological processes. The report of Cochrane et al. [111] showed that overexpression of *Phytogbs1* in *H*. *vulgare* under hypoxic conditions led to a higher ATP/ADP ratio. On the other hand, *Phytogbs1* knockdown correlated with an increase in protein R-SNO as well as ADH and GSNOR activities. Barley plants with *Phytogbs1* overexpression were the only ones that grew in normoxia, and their seeds germinated under hypoxia. These data also suggested the role of PGB in NO capture, which affects the energy state of plants under low oxygen. Moreover, several reports indicated different links between the PGB-NO cycle and other factors during germination. Oxygen deficiency in the aleurone layers of germinating barley seeds and seedlings led to the expression of *Phytogbs1* and *Phytogbs3* genes. Phytoglobins were induced in the aleurone layers through an increase in gibberellic acid concentration and α-amylase activity. In turn, ABA promoted seed dormancy through changes in the expression of alcohol dehydrogenase (ADH) genes. It has been suggested that the PGB-NO cycle regulates the ethanol fermentation pathway during germination. Therefore, the PGB-NO cycle is an alternative pathway for obtaining energy in hypoxia. In addition, a study by Kumari et al. [112] showed the participation of the PGB-NO and AOX cycles in the germination and growth of *Oryza sativa* plants.

## 4. The Role of NO in PTMs

The biological function of NO is also related to PTMs. In this review, we would like to describe three protein modifications in which the effects of NO are clearly visible, namely, R-SNO, nitration, and the phosphorylation of proteins.

### 4.1. S-Nitrosylation

**Figure 3:**
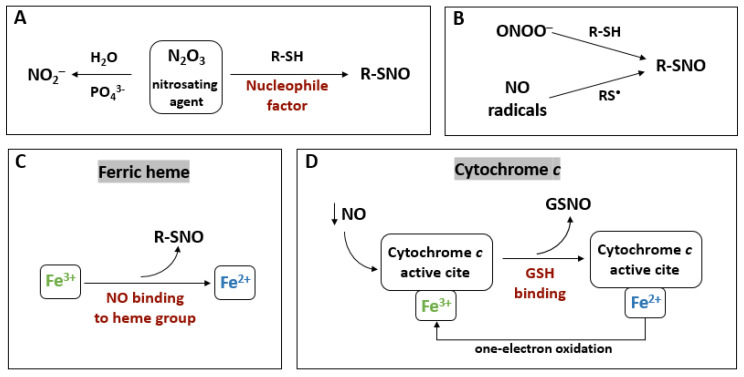
*S*-nitrosothiol generation pathways. (**A**) An interaction of thiolates (R-SH) with NO autooxidation products. Dinitrogen trioxide (N_2_O_3_) in the presence of a nucleophilic agent (other than water) and R-SH interaction leads to *S*-nitrosylation (R-SNO). (**B**) A radical pathway of R-SNO. An interaction between peroxinitrite (ONOO^−^) with R-SH or NO radicals with thiyl radicals (RS^•^) induces R-SNO. (**C**) R-SNO via ferric transnitrosation in haem proteins. The reduction of Fe^3+^ to Fe^2+^ during NO binding to heme group. (**D**) The generation of SNOs via ferric transnitrosation in cytochrome *c*. At low NO concentration, cytochrome *c* can bind reduced glutathione (GSH). This reaction is accompanied by a reversible ferric reduction and S-nitrosoglutathione (GSNO) synthesis.

The most important NO-dependent protein modification is R-SNO. This non-enzymatic reversible reaction leads to the covalent addition of NO to the sulfur group of cysteine residues in the target protein. As a result of R-SNO, SNOs are formed [113,114]. In plants, R-SNO is reported to be involved in various physiological processes such as responses to environmental stresses [115,116,117] and phytohormone signaling [118,119]. In this case, the formation of SNOs is possible via nitrogen oxides (N_x_O_y_) and complexes of metals with NO, SNO, NO_2_^−^, and ONOO^−^ [114,120]. The work of Smith and Marletta [121] systematized the knowledge about the formation of SNOs through various mechanisms. The first of the paths of SNOs generation is the interaction of thiolates (R-SH) with NO autooxidation products. For example, the interaction of reactive N_2_O_3_ with R-SH led to SNOs via R-SNO [121,122]. N_2_O_3_ is confirmed as an effective nitrosating agent only in the presence of a nucleophilic agent other than water. The reaction of water with N_2_O_3_ results in hydrolysis (in the presence of phosphate) to nitrite (Figure 3A).

A second method of SNOs formation is through a radical pathway in which thiyl radicals are engaged. SNOs are generated through a NO-dependent mechanism, for example through the interaction of ONOO^−^ with cysteine residues, or through the interaction between NO radicals and Cys thiyl radicals [56] (Figure 3B).

A third possible mechanism for SNOs generation is called metal transnitrosation (via one-electron oxidation). This pathway leads to the generation of the thionitroxide radical by combining NO with thiol. It has been shown that iron (Fe^3+^) in the protein heme group can bind via NO. This interaction triggers the formation of SNOs via the reduction of Fe^3+^ to Fe^2+^ [56,121] (Figure 3C).

The formation of SNO via the ferric cytochrome *c*-dependent way is based on the binding of GSH to the active cytochrome *c* [122,123]. This interaction leads to the slow reduction of iron and the release of GSNO (Figure 3D). In addition, it is believed that the most effective reduction of cytochrome *c* by GSH occurs at low NO concentration. Thus, the concentration of NO can control the cytochrome *c* reduction as well as the generation of GSNO. Continuous oxidation of cytochrome *c* (e.g., via mitochondrial complex IV) is probably required to maintain the ability to reduce iron [122]. An understanding of the pathways of GSNO formation is important due to the fact that this SNO is the main cellular reservoir of NO in plants, and it is involved in NO signaling [124].

In addition, R-SNO can mediate reversible transnitrosation between SNO and thiols (e.g., cysteine) [121,125]. In the case of Cys-Cys transnitrosation, a nucleophilic thiol attack of the NO residue in the SNO is observed. Consequently, this reaction leads to the formation of nitroxyl disulfide, which is an intermediate transnitrosation product. Data obtained with nuclear magnetic resonance spectroscopy [126] and mass spectrometry [127] revealed that the transformation does not lead to the formation of free NO or its protonated form.

### 4.2. Protein Nitration

**Figure 4:**
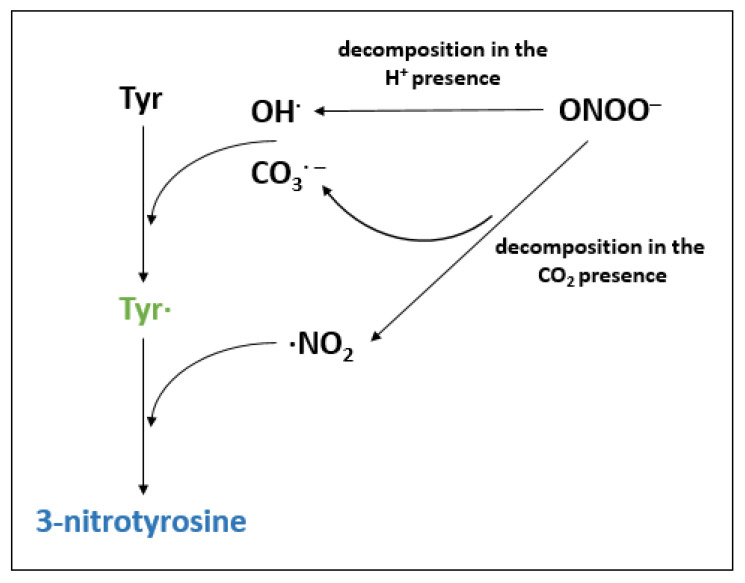
Two-step nitration of tyrosine (Tyr) in the presence of decomposition products of peroxynitrite (ONOO^−^): OH^•^, CO_3_^•−^, and ^•^NO_2_. In the first step, the interaction of OH^•^/CO_3_^•−^ with tyrosine takes place, resulting in the formation of a tyrosyl radical (Tyr^•^). In the second reaction, Tyr^•^ and ^•^NO_2_ form 3-nitrotyrosine.

Another example of NO-dependent PTM is nitration. The effect of this modification is the addition of a nitro group (−NO_2_) to tyrosine (Tyr), tryptophan (Trp), methionine (Met), or cysteine residues. In animal and plant cells, this modification affects not only proteins, but also nucleic acids, fatty acids, and oligonucleotides. Previous data focused primarily on the understanding of the formation of nitro-tyrosine [128,129,130]. Tyrosine nitration is a non-enzymatic, two-step process (Figure 4), based on the covalent modification of ortho-carbon in Tyr’s aromatic ring. In the first step, the tyrosyl radical (Tyr^•^) is formed through the oxidation of the aromatic ring of Tyr, due to the influence of oxidants (hydroxyl (OH^•^) and carbonate (CO_3_^•−^) radicals) formed as a result of the decomposition of ONOO^−^. Next, Tyr^•^ interacts with ^•^NO_2_ (formed through the decomposition of ONOO^−^ in the presence of CO_2_), which results in the formation of 3-nitrotyrosine [128,130,131]. Due to the fact that this mechanism affects the sites of free radical formation, nitrated proteins are usually located close to the compartments of their formation due to the short biological half-life of nitrogen radicals, e.g., ONOO^−^ [132,133,134]. In addition, only susceptible Tyr surrounded by amino acid residues with the appropriate redox potential can be nitrated in proteins [135]. The research of Bayden et al. [136] focused on the three-dimensional structure of nitro-tyrosine-containing proteins under oxidative stress. The results indicated the significant role of the acidic and basic residues adjacent to Tyr. The distance to the nearest heteroatom in the charged side chain of the adjacent amino acid is believed to correspond to the distance needed to form a hydrogen bond between Tyr and that amino acid. Furthermore, nitration is probably hindered in the absence of a site for attachment of the nitro group [136]. The addition of NO_2_ to Tyr leads to a decrease in the isoelectric point of the protein. It may affect the availability of Tyr to participate in the electron transfer reaction and lead to protein conformation changes [137,138,139]. In most cases, nitration is an irreversible reaction that can usually cause a loss or inhibition of protein function [137,140,141]. Increasing the hydrophobicity of the Tyr residue after nitration also promotes conformational changes [131]. Nitration may affect cell signaling through regulating the level of Tyr phosphorylation. It is hypothesized that increasing Tyr phosphorylation may regulate the ONOO^−^-mediated nitration of peptides [142,143]. In addition, studies by Galetskiy et al. [143] showed the simultaneous effect of phosphorylation and nitration on the stability of photosystems I and II in Arabidopsis plants under high light stress. Previous studies have focused particularly on understanding the basis of the contribution of Tyr nitration to the development and response to abiotic/biotic stress in plants [125,144,145,146]. An important widely discussed problem is the link between nitration and other redox PTMs (e.g., R-SNO) in the signaling network and redox status under stress conditions [144,147,148]. The research of Chaki et al. [149] showed that mechanical wounding induces accumulation of GSNO and simultaneously an inhibition of NOS-like and GSNOR activities. Researchers indicated that ONOO^−^ formation promoted R-SNOs via Tyr nitration. It was possible through the conversion of GSNO in the presence of O_2_^−^ to radical glutathione and ONOO^−^. The described pathway of nitration is a component of signaling under biotic stresses.

### 4.3. Phosphorylation

Increased NO content in tissues may trigger NO-signaling cascades dependent on phosphorylation [150] through the activation of specific protein kinases (PKs), which leads to the final phosphorylation of target factors. Phosphorylation may trigger activation of the appropriate signaling pathways, which causes the expression of genes involved in physiological responses to stimuli. The NO-dependent PKs most frequently discussed in the literature are sucrose non-fermenting 1-related protein kinase 2 (SnRK2) [151], mitogen-activated protein kinases (MAPKs) [152], and calcium-dependent kinases (CDPKs) [153]. So far, the mechanisms through which NO affects individual PKs are not fully understood.

SnRK2 is a positive regulator of ABA signal transduction [154]. SnRK2 requires prior autophosphorylation to fulfill its catalytic function. Then, its activity leads to the phosphorylation of transcription factors—the binding factor for ABA-responsive elements (ABF), which in turn contributes to the induction of ABA-induced genes [155,156]. Studies by Wang et al. [157] showed a putative NO influence on SnRK2s. NO can inhibit the activation of mentioned kinases via their S-nitrosylation by GSNO, for example inactivation of OST1/SnRK2.6 kinases under the drought was shown. This protein is involved in ABA-dependent stomatal closure [157]. *S*-nitrosylation of the Cys-127 residue at the SnRK2 catalytic site led to inhibition of kinase activity, and GSNOR knockout mutants showed impaired stomatal closure. It indicates that NO accumulation in guard cells could cause S-nitrosylation of SnRK2.6. Moreover, another report [158] confirmed the negative role of NO in the regulation of ABA signaling during Arabidopsis seed germination and growth of seedlings via *S*-nitrosylation of SnRK2.3 and SnRK2.3 caused by the NO donor—sodium nitroprusside (SNP).

In addition, *Cucumis sativus* research showed the involvement of CDPKs in auxin signaling. Studies have shown that CDPK engaged in the formation of adventitious roots of *C*. *sativus* induced by indole-3-acetic acid (IAA) and NO. These results suggested that in the presence of SNP and IAA, auxin signal transduction was triggered by the activation of phosphorylated CDPK [159].

## 5. Crosstalk between NO, ROS, and Phytohormones

Maintaining redox balance is a key factor in the functioning of plant cells under optimal and stress (abiotic/biotic) conditions. Table 1 summarizes the interactions between NO, ROS, and phytohormones in plants. ROS are a heterogeneous group of chemicals; therefore, different molecules can affect cellular processes in different ways depending on their redox potential, concentration in cellular compartments, and individual chemical characteristics [20,160]. For example, the low endogenous content of superoxide anions H_2_O_2_, and OH^•^ may have a positive effect on physiological processes in plants [161]. On the other hand, overproduction of ROS may lead to secondary oxidative stress [162]. Therefore, the presence of non-enzymatic antioxidants (e.g., ascorbic acid (AsA), GSH, phenolic compounds) as well as increased activity of antioxidant enzymes (e.g., superoxide dismutase (SOD), catalase (CAT), peroxidase (POD), ascorbate peroxidase (APX), glutathione reductase (GR), and glutathione *S*-transferase (GST)) allows to control the oxidative state and maintain redox homeostasis through converting ROS to non-toxic molecules [161,163,164,165,166,167,168,169,170,171].

Compounds formed as a result of ROS enzymatic transformations may participate in NO metabolism. This ROS/RNS crosstalk may lead to signal transduction through redox-based modifications [61]. The synthesis and conversion of H_2_O_2_ affect NO metabolism. The reaction catalyzed by SOD leads to the formation of H_2_O_2_. Next, H_2_O_2_ is converted by CAT or glutathione peroxidase (GPX) to water and oxygen. GPX catalyzes the reduction of different hydroperoxides via oxidation of GSH into GSSG [164,200]. GSSG conversion by GR cause an increase in GSH content [201]. Consequently, in the presence of oxygen, NO can interact with GSH to form GSNO. [86,88]. Crosstalk between these signaling molecules has been shown to play an essential role in the development of responses to abiotic factors through the regulation of gene expression [161,202,203]. In addition, the role of NO and ROS as mediators of plant acclimatization to diseases and herbivores has been widely discussed [172]. NO and ROS were shown to affect the non-expressing pathogenesis-related gene 1 (NPR1) through regulating the S-nitrosylation of this protein. NPR1 activity was associated with systemic acquired resistance (SAR) [172,173,174].

Moreover, the increased concentration of ROS and NO is an early modulator of the development of hypersensitivity response (HR) and PCD in infected tissues [177,178,179]. Increased content of NO and ROS is also observed during the attacks of herbivores. Studies revealed that high GSNOR activity during pathogenesis leads to GSNO formation, creating a reservoir of NO, which makes GSNOR a key enzyme in pathogen resistance [172,175,176]. Further data suggest that GSNOR activity affects the metabolism of jasmonic acid (JA) and ethylene (ET) during insect feeding [204].

NO can interact with ABA-related signal transduction. The pyrabactin resistance/pyrabactin resistance-like/regulatory component of ABA receptors (PYR/PYL/RCAR) play a vital role in the ABA-dependent responses of plants to external or internal stimuli [205] and can be inactivated via the nitration of Tyr residue triggered by NO/ONOO^–^. Receptor nitration leads to its polyubiquitylation and degradation by the proteasome [181]. This mechanism of inhibition of ABA signaling by NO occurs with high concentrations of NO and ROS in tissues. In addition, ABA signal transduction is limited by the S-nitrosylation of Cys153 in the ABA-insensitive 5 (ABI5) transcription factor [182]. The results showed that S-nitrosylated ABI5 interacted with the ubiquitin-proteasome system (UPS) through E3 ligases based on CULLIN4 and KEEP ON GOING. Studies on the Arabidopsis *nia1nia2* mutant (lack of NO production by NR) showed an upregulation of *RCAR1*, *RCAR11,* and *RCAR12* only in the presence of NO [63].

The positive effect of CDPK phosphorylation on lateral/primary root formation is an example of the involvement of NO in auxin signaling [183,184]. There is also evidence of NO and IAA crosstalk with a positive effect on nodule formation [206]. Overproduction of IAA by *Sinorhizobium meliloti* (in the presence of NO) has been shown to promote nodulation in *Medicago* species and enhance lateral root formation. On the other hand, studies using *O*. *sativa* seedlings under cadmium and arsenic stresses showed the interaction of NO and IAA (and its precursor—indole-3-butyric acid) in preventing the negative impact of heavy metals [185]. It was found that the decreasing content of IAA in the roots of *O*. *sativa* was accompanied by the protective role of NO against the negative effects of the presence of Cd and As. It was shown that the presence of an NO donor (SNP) led to increased production of ROS, which consequently oxidized IAA. A similar result was obtained in experiments on *Medicago trancatula* under Cd stress. It was found that Cd treatment reduced root growth and NO accumulation. However, an increased level of ROS in the roots of *M*. *trancatula* was observed [186].

The use of the NO scavenger 2-4-carboxyphenyl-4,4,5,5-tetramethylimidazoline-1-oxyl-3-oxide (cPTIO) inhibited NR and NOS-like activities. With external NO application, root growth was improved through preventing auxin degradation, inhibiting IAA oxidase, and accumulating antioxidants (proline and GSH). Terrile et al. [207] pointed out a significant problem regarding the lack of molecular knowledge on the interaction of NO and IAA in root growth modulation. Research focused on auxin receptors—transport inhibitor response 1/auxin signaling f-box (TIR1/AFB), which cooperate in the degradation of the auxin repressor auxin/IAA. The obtained results suggested that the S-nitrosylation of Cys140 in Tir1 improved the interaction of TIR1 with IAA, auxin/IAA degradation, and the expression of IAA-dependent genes.

Salicylic acid (SA) participates in a number of physiological processes such as seed germination, growth, and flowering. It increases the activity of photosynthesis [208,209]. Nevertheless, the main function of SA is participation in response to environmental stresses [10,210,211,212]. In response to a microbial pathogen, the systemic acquired resistance (SAR) mechanism is activated. It is strictly regulated by Ca^2+^ and nucleotide-gated cyclic ion channels [2,213,214], and hence, increasing Ca^2+^ content during plant defense has been observed [215]. Moreover, SA can be reversibly converted to the methylated form—methyl salicylate (Me-SA). It is considered that Me-SA in contrast to SA is involved in response to biotic stresses. Further transformations of Me-SA, e.g., glycosylation, enable maintenance of the function of methylated salicylic acid and proper development of the SAR [216,217]. In addition, it is indicated that exogenous NO treatment may lead to the changes in SA concentration during the induction of defense genes expression in tobacco [34]. Studies on the *A. thaliana nia1nia2* mutant have shown that SA may favor NO generation through NOS-like activity [187]. This observation was also confirmed by the identification of the signaling component of SA-mediated pathway—calcium and casein kinases, which are involved in triggering NOS-like activity by SA. It is reported that NO and ROS may act in cooperation with SA to activate defensive redox signaling [218]. The molecular regulation of NO-SA crosstalk is related to the expression of NPR genes during SAR [188,189]. Under stress conditions, signal induction by NPR contributes to the stimulation of SA-NO response development. Optimal environmental conditions inactivate NPR through induction of its oligomerization. Oligomers are re-accumulated with increasing SA content [157,188,219]. The oligomerization status of NPR depends on S-nitrosylation via NO, which is regulated by SA [190,220,221]. On the other hand, GSNO treatment of *A. thaliana* plants triggered crosstalk of the NO- and SA-dependent response during *Pseudomonas* infection [190,191]. The presence of an NO donor mediated the accumulation of NPR1 in the nucleus and ensured the activation of pathogenesis-related (PR) genes. An increase in SA content was caused by a sudden change in GSH concentration, which involves SA synthesis. In addition, MAPKs also participate in the development of plant resistance to pathogens. NO may cooperate with SA in modulating the activity of SA-induced protein kinase (SIPK),which is a member of MAPKs [192,193].

Jasmonic acid is classified as a lipid signaling molecule associated not only with plant growth, but especially with plant resistance to biotic and abiotic stresses. Jasmonic acid has several derivatives which are referred to as jasmonates (JAs) [222,223]. One of the major forms is methyl jasmonate (Me-JA). Increasing JA conversion to volatile Me-JA enhances resistance against pathogens and abiotic factors through influencing the activity of defense-related and antioxidant enzymes. Moreover, Me-JA takes part in the initiation of pathogenesis-related gene expression [224,225]. The most commonly reported JA interaction is JA-SA crosstalk in defense responses to pests and pathogens [226,227]. The above-mentioned NPR1 is also involved in JA-SA signaling. Modification of NPR1 and the TGACG-binding (TGA) transcription factor by SA has been found to mediate the downregulation of JA-dependent genes [194]. It is still unclear how NPR1 inhibits the expression of genes involved in the JA-SA-NO crosstalk. One possible mechanism is NPR1 oligomerization via S-nitrosylation [196,228]. Recent data indicate the involvement of the transcription complex (basic-helix-loop-helix MYC2 transcription factors and mediator complex subunit 25) in interaction with NPR1 (activated by SA and indirectly by NO) in the suppression of transcription of JA-dependent genes [195]. The direct effect of NO on JA biosynthesis was shown via transcriptome analysis, where NO mediated the induction of the expression of JA synthesis enzymes (lipoxygenase 2 (LOX) and 12-oxophytodienoate reductase (OPR)) [196,197]. In addition, experimental data indicated that another enzyme involved in JA synthesis, allene oxide cyclase (AOC), was inhibited by NO via S-nitrosylation [196,198]. NO-JA crosstalk plays a role in the response to abiotic stress. In *T*. *aestivum* plants under drought, JA treatment caused the release of NO molecules which led to the activation of the ascorbate-glutathione cycle and thus enabled plant growth in unfavorable conditions [49,199].

## 6. In Search of NO-Dependent Defense Mechanisms during Infection with Herbivorous Ecdysozoa Species

Ecdysozoa is a group of invertebrates characterized by molting [229,230]. The role of NO in defense mechanisms during infestation with nematodes and arthropods (Arachnida, Insecta) is discussed below.

### 6.1. Nematodes

NO plays a significant role in the response to biotic stresses, including the attack of nematodes on plant roots. The results obtained on *Solanum lycopersicon* plants infected with the root-knot nematode *Meloidogyne incognita* (RKN) (Nematoda: Heteroderidae) indicated the role of the interaction of NO, JA, and protease inhibitors in plant defense against RKN [231]. These findings were confirmed through analysis of the expression of genes from NO and JA synthesis pathways. Researchers showed that due to RKN infection, transcript levels of genes related to NO and JA were significantly increased. In addition, the effect of exogenous applications of JA and SNP on the reproductive capacity of RKN was investigated [231]. The presence of JA and SNP led to a reduction in the number of RKN eggs and partial inhibition of nematode growth. Moreover, an improvement in photosynthesis in comparison to the infected plants was observed. RKN infection affects not only the inhibition of root growth, but also net photosynthesis rate. Therefore, it could indirectly limit roots formation through lowering the concentration of photosynthesis products. RKN infection also resulted in increased electrolyte leakage and lipid peroxidation in the roots. cPTIO treatment reduced the JA-related defense response to the RKN. On the other hand, the exogenous application of SNP and JA during RKN infection was accompanied by the induction of protease inhibitor 2 gene expression, which may be of paramount importance because the silencing of the protease inhibitor 2 gene contributed to greater susceptibility to RKN infection [231].

Melillo et al. [232] showed the generation of NO and ROS in a *S*. *lycopersicon* variety resistant to RKN. These data suggested that rapid NO accumulation in tomato tissues after RKN invasion is a result of NOS-like activity. In infected roots, the H_2_O_2_ content increased rapidly in the first 24 h post-inoculation (hpi). The simultaneous presence of H_2_O_2_ and NO caused PCD in infected tomato roots, thus contributing to developing a defense response against RKN [232,233]. In turn, another study showed the highest production of NO via NOS-like activity at 12 hpi in response to RKN [234].

In addition, studies of *Pinus thunbergii* response to the pine wood nematode *Bursaphelenchus xylophilus* (Nematoda: Aphelenchoididae) infection also showed the cooperation of NO and H_2_O_2_ [235]. Changes in the concentration of NO occurred at an earlier stage (8 hpi) than H_2_O_2_ (12 hpi). The SNP treatment triggered NO synthesis via NOS-like activity (not via NR) in infected *P. thunbergii* plants. Therefore, the crosstalk between NO and H_2_O_2_ may be considered as part of the defense mechanism in the initial response of *P. thunbergii* to the invasion of nematodes [235].

Our previous experiments showed that a beet cyst nematode *Heterodera schachtii* (Nematoda: Heteroderidae) infestation led to the production of NO and ONOO^−^ in infected *A. thaliana* roots. These observations were accompanied by an increase in abundance of *S*-nitrosylated and nitrated proteins. The activity of GSNOR was reduced at 3 and 15 days post-inoculation (dpi) and enhanced at 7 dpi in infected roots, whereas the *GSNOR1* transcript level was increased over the entire examination period. The GSNOR level was enhanced in infected roots at 3 dpi and 7 dpi, but at 15 dpi, did not differ between uninfected and infected roots. The GSNOR was observed in plastids, mitochondria, the cytoplasm, as well as in the endoplasmic reticulum and cytoplasmic membranes [236].

### 6.2. Insects

Insect infection leads to the development of defense mechanisms involving NO. Cytochemical localization of NO performed in *Pisum sativum* plants infested with the pea aphid *Acyrthosiphon pisum* (Hemiptera: Aphididae) revealed the highest NO fluorescence signal at 48 hpi. Aphid invasion triggered the synthesis of other molecules related to the response to biotic stress (JA, ET, SA, H_2_O_2_). At 48 hpi, an increase in the content of ET and NO in the leaves was observed. On the other hand, intense SA synthesis occurred only at 74 and 96 hpi [237].

The work of Woźniak et al. [238] showed that the use of exogenous NO donors contributed to the defensive responses of *P*. *sativum* against *A*. *pisum*. The treatment of infected *P*. *sativum* plants with SNP and GSNO led to a reduction in superoxide anion level at 48 and 72 hpi and an induction of phenylalanine ammonialyase gene expression [238].

Research by Xu et al. [239] showed that NO mediated the suppression of the JA defense during a silverleaf whitefly *Bemisia tabaci* (Hemiptera: Aleyrodidae) invasion. Infested tobacco plants were characterized by a high accumulation of NO. Compared with water-treated plants, which were considered 100%, 71% of *B*. *tabaci* adults settled on the plants treated with SNP, whereas only 29% of adults settled on the plants treated with cPTIO. In addition, the use of SNP favored the acceleration of nymph maturation. On the other hand, after the use of cPTIO, a disturbance in the development cycle of nymphs was observed [239].

The cotton bollworm *Helicoverpa armigera* (Lepidoptera: Noctuidae) is a pest that feeds on the chickpea *Cicer arietinum*. A study comparing the defense mechanisms of sensitive and resistant *C. arietinum* varieties showed differences in chickpea responses against *H. armigera* invasion [240]. The sensitive variety was characterized by a decrease in the activity of antioxidant enzymes and reduced phenols, NO, H_2_O_2,_ and trypsin inhibitor levels. In the resistant variety, all above parameters were constitutively increased [240].

The results obtained from the study of susceptible and resistant varieties of *T*. aestivum to the Russian wheat aphid *Diuraphis noxia* (Hemiptera: Aphididae) showed that, in the resistant variety, infection led to earlier accumulation of NO [241]. In addition, an engagement of NR I NiR in the production of NO during infestation was observed. It was shown that NO regulated the response to *D. noxia* through induction of *β*-1,3-glucanase and POX [241].

*GSNOR* gene silencing in *Nicotiana attenuate* led to greater susceptibility to invasion by the Carolina sphinx *Manduca sexta* (Lepidoptera: Sphingidae) [204], which was accompanied by reduced JA and ET contents. However, no inhibition of MAPK activity was observed, and the content of trypsin inhibitors decreased in the infected plants. Researchers have speculated that GSNOR may be involved in JA-dependent defense [204].

An analysis of the effect of the exogenous application of an NO donor in species of forage grasses (*Brachiaria ruziziensis*, *Pennisetum purpureum*, and *Digitaria* sp.) attacked by *Mahanarva spectabilis* (Hemiptera: Cercopidae) showed an increased concentration of phenols in the infested plants. However, increased phenol content did not contribute to the inhibition of the development cycle of *M*. *spectabilis* nor resistance to this pest [242].

A study of two *O*. *sativa* cultivars differing in their resistance to the brown planthopper (BPH) *Nilaparvata lugens* (Hemiptera: Delphacidae) showed elevated levels of NO in the leaves of both cultivars after infection and in the roots of the resistant cultivar. Scientists hypothesized that NO synthesis in response to *N*. *lugens* depends on NOS-like activity. In addition, treatment with an exogenous NO donor reduced plant mortality due to BHP infestation and caused the expression of drought-related genes (*OsLea3-1* and *OsP5CS1*) [243].

### 6.3. Arachnids

The literature on NO-dependent responses of plants infested with arachnids is extremely limited [175,244]. In plants infested with arachnids, the involvement of ROS metabolism [245,246,247], antioxidant enzymes [246,248], phytohormone interactions [249,250], and transcriptomic [251,252] and proteomic changes [253] have been studied. Nevertheless, despite the proven strong links between NO and the response to biotic stresses, there is no direct evidence in the literature.

### 6.4. Are There Common Patterns of NO-Dependent Defensive Responses against Ecdysozoa Species Infestation?

Table 2 shows the contribution of NO to plant responses to nematode and insect infestations. These examples show that nematode attack responses are related to changes in NO and GSNO contents. Furthermore, NO production in response to infections is usually due to NOS-like activity rather than NR activity. Moreover, infections with nematodes lead to interactions between NO and JA.

In turn, NO-dependent responses to insects involve an interaction between NO and JA, ET, SA, and H_2_O_2_. Our review strongly points to the need for further research on NO interactions between host plants and Ecdysozoa parasites, especially arachnids.

## 7. Conclusions

Recent studies have shown the multifunctional role of NO in plants; however, there are still many questions regarding the involvement of NO in a number of biochemical/physiological processes and the regulation of NO-dependent gene expression. Considering crosstalk between NO and ROS, ABA, IAA, SA, and JA, multiple response pathways are involved in plant defense responses to biotic stressors. This review clearly shows that despite the many available data points in the literature, it is still difficult to determine the mechanisms of NO action in response to, e.g., the attack of herbivorous Ecdysozoa. Moreover, there is a need for holistic research showing the contribution of NO in response to a combination of stress factors. Although the various aspects of NO multifunctionality are relatively well known, most studies focus on isolated stresses and key developmental moments, which do not reflect the role of NO in plants under field conditions. Further studies of NO need to be conducted to develop new cultivars that are resistant/tolerant to biotic and abiotic stresses.

## Figures and Tables

**Table 1:** Crosstalk between nitric oxide (NO), reactive oxygen species (ROS), and phytohormones: abscisic acid (ABA), indole-3-acetic acid (IAA), salicylic acid (SA), and jasmonic acid (JA). Abbreviations: mitogen-activated protein kinases (MAPK); programmed cell death (PCD); *S*-nitrosylation (R-SNO); non-expresser of pathogenesis-related gene 1 (NPR1); systemic acquired resistance (SAR); *S*-nitrosoglutathione reductase (GSNOR); *S*-nitrosoglutathione (GSNO); hypersensitive response (HR); pyrabactin resistance/pyrabactin resistance-like/regulatory component of ABA receptors (PYR/PYL/RCAR); ubiquitin-proteasome system (UPS); non-fermenting 1-related protein kinase 2 (SnRK2); ABA-insensitive 5 (ABI5); calcium-dependent kinases (CDPKs); sodium nitroprusside (SNP); nitric oxide synthase (NOS); nonexpresser of PR genes (NPR); pathogenesis-related (PR); SA-induced protein kinase (SIPK); TGACG-binding (TGA) transcription factor; lipoxygenase 2 (LOX2); 12-oxophytodienoate reductase (OPR); allene oxide cyclase (AOC).

NO Crosstalk	Influence	Effect	References
ROS	NO-H_2_O_2_ modulation of transcription factors	putative R-SNO and cysteine residue oxidation	[172]
	NO-H_2_O_2_—MAPK phosphorylation	PCD activation	[172]
	R-SNO of NPR1 protein	SAR activation	[172,173,174]
	GSNO production by GSNOR	presence of NO reservoir under pathogen attack	[172,175,176]
	HR gene expression regulation	HR and PCD	[177,178,179]
ABA	induction of (+)-ABA 8′-hydroxylase expression	ABA signaling inhibition (breaking seed dormancy)	[180]
	Tyr nitration of PYR/PYL/RCAR	PYR/PYL/RCAR degradation by UPS	[181]
	R-SNO of Cys residue of SnRK2	inhibition of SnRK2	[157,158]
	R-SNO of Cys residue of ABI5	degradation of ABI5 by UPS	[182]
IAA	phosphorylation of CDPK	lateral/primary root growth	[183,184]
	IAA-overproduction by *Sinorhizobium meliloti* (in presence of NO)	nodulation in *Medicago* species	[185]
	production of ROS	oxidized IAA	[186]
SA	induction of defense genes expression	regulation of SA level during biotic stress	[34]
	induction of NOS-like activity	NO synthesis	[187]
	molecular regulation of *NPR* gene expression	induction of SAR via NO-SA crosstalk	[188,189]
	accumulation of NPR1	activation of *PR* genes	[190,191]
	modulation of SIPK	development of resistance to pathogen	[192,193]
JA	NPR1 and TGA modifications	suppression of JA-dependent genes	[194]
	interaction of NPR1 and basic-helix-loop-helix transcription factors MYC2-mediator complex subunit 25	suppression of JA-dependent genes	[195]
	induction gene expression of *LOX2* and *OPR*	JA synthesis	[196,197]
	R-SNO of AOC	decreased JA synthesis	[196,198]
	activation of ascorbate-glutathione cycle	plant growth improvement under drought	[49,199]

**Table 2:** Nitric oxide (NO)-dependent responses against infestation with nematodes and insects. Abbreviations: jasmonic acid (JA); programmed cell death (PCD); reactive oxygen species (ROS); nitric oxide synthase (NOS); reactive nitrogen species (RNS); *S*-nitrosylation (R-SNO); *S*-nitrosoglutathione reductase (GSNOR); salicylic acid (SA); ethylene (ET); nitrate reductase (NR); nitrite reductase (NiR).

Parasite	Plant	Response	References
Nematodes			
*Meloidogyne incognita*	*Solanum lycopersicon*	increased expression of NO- and JA-induced genes	[231]
*Meloidogyne incognita*	*Solanum lycopersicon*	NO-H_2_O_2_ crosstalk, PCD activation	[232]
*Meloidogyne incognita*	*Solanum lycopersicon*	NO-ROS crosstalk, increased NOS-like activity	[234]
*Bursaphelenchus xylophilus*	*Pinus thunbergii*	increased NOS-like activity	[235]
*Heterodera schachtii*	*Arabidopsis thaliana*	alteration in the level of RNS, protein R-SNO and nitration, and GSNOR	[236]
Insects			
*Acyrthosiphon pisum*	*Pisum sativum*	interconnection of NO production with JA, ET, SA, H_2_O_2_ synthesis	[237]
*Acyrthosiphon pisum*	*Pisum sativum*	restriction of aphids’ reproduction	[238]
*Bemisia tabaci*	*Nicotiana tabacum*	suppression of JA-defense responses and favoring *B. tabaci* reproduction	[239]
*Helicoverpa armigera*	*Cicer arietinum*	changes in antioxidants enzymes, NO, H_2_O_2_, phenols and trypsin inhibitor	[240]
*Diuraphis noxia*	*Triticum aestivum*	changes in NR and NiR activities, NO-dependent induction of β-1,3-glucanase and peroxidase	[241]
*Manduca sexta*	*Nicotiana attenuata*	GSNOR interconnection with NO- and JA-dependent responses	[204]
*Mahanarva spectabilis*	*Brachiaria ruziziensis, Pennisetum purpureum* and *Digitaria* sp.	increased content of phenols, lack of inhibition of pest development cycle	[242]
*Nilaparvata lugens*	*Oryza sativa*	increased NO content in resistant cultivar, increased expression of genes related to drought response	[243]

## Data Availability

Not applicable.
